# Crystal structure of tricarbon­yl(*N*-di­phenyl­phosphanyl-*N*,*N*′-diisopropyl-*P*-phenyl­phospho­nous di­amide-κ^2^ *P*,*P*′)cobalt(I) tetra­carbonyl­cobaltate(−I) toluene 0.25-solvate

**DOI:** 10.1107/S1600536814024908

**Published:** 2014-11-19

**Authors:** Laura Dura, Anke Spannenberg, Torsten Beweries

**Affiliations:** a Leibniz-Institut für Katalyse e.V. an der Universität Rostock, Albert-Einstein-Strasse 29a, 18059 Rostock, Germany

**Keywords:** crystal structure, cobalt, phosphine ligand, metallacycle

## Abstract

The asymmetric unit of the title compound, [Co(C_24_H_30_N_2_P_2_)(CO)_3_][Co(CO)_4_]·0.25C_7_H_8_, consists of two crystallographically independent cations with similar conformations, two anions, and one-half of a toluene mol­ecule disordered about an inversion centre. In the cations, a Co/P/N/P four-membered slightly bent metallacycle is the key structural element. The pendant NH group is not coordinated to the Co^I^ atom, which displays a distorted trigonal–bipyramidal coordination geometry. Weak inter­ionic hydrogen bonds are observed between the NH groups and a carbonyl group of the tetrahedral [Co(CO)_4_]^−^ anions.

## Abstract 2

The title compound {Co(CO)_3_[Ph_2_PN(*i*-Pr)P(Ph)N(H)*i*-Pr]}[Co(CO)_4_] is an ionic species consisting of a Co(CO)_3_[Ph_2_PN(*i*-Pr)P(Ph)N(H)*i*-Pr] cation and a [Co(CO)_4_] anion.

## Chemical context   

Reaction of the PNPNH ligand *N*-(diphenylphosphanyl)-*N*,*N′*-diisopropyl-di­amino­phenyl­phosphine, Ph_2_PN(*i*-Pr)P(Ph)N(H)*i*-Pr, with the cobalt precursor Co_2_(CO)_8_ was performed to prepare a noble-metal-free catalyst for light-driven water reduction to produce hydrogen. These compounds are attractive in terms of environmental acceptability as well as for economic reasons. Several very active inter­molecular water-reduction systems using 3*d* metal complexes as catalytically active centres are known, examples include work on iron (*e.g.* Mejía *et al.*, 2013 ▶), nickel (*e.g.* Zhang *et al.*, 2011 ▶) and cobalt (*e.g.* Tong *et al.*, 2014 ▶) complexes. It is likely that the NH group of the ligand and the Co atom cooperate in the proton-reduction process as has been reported for other water-reduction complexes (Han *et al.*, 2012 ▶). We found that, apart from the previously described catalytically active dinuclear CO-bridged product Co_2_(CO)_6_(PNPNH) (Hansen *et al.*, 2013 ▶), an ionic complex is also formed in this reaction. Both complexes can be separated by fractionated crystallization from toluene. It should be noted that in solution, the title compound is rapidly converted into the neutral dinuclear species Co_2_(CO)_6_(PNPNH) and therefore the IR and NMR spectra were measured only from freshly prepared samples.
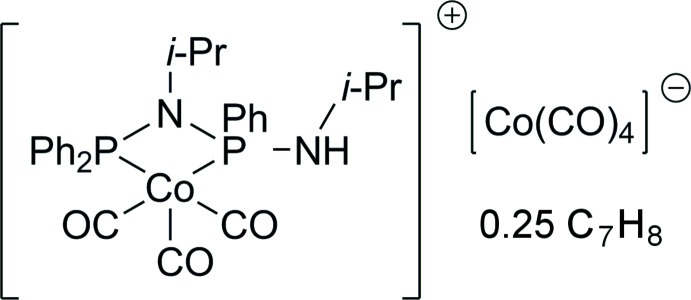


## Structural commentary   

**Figure 1:**
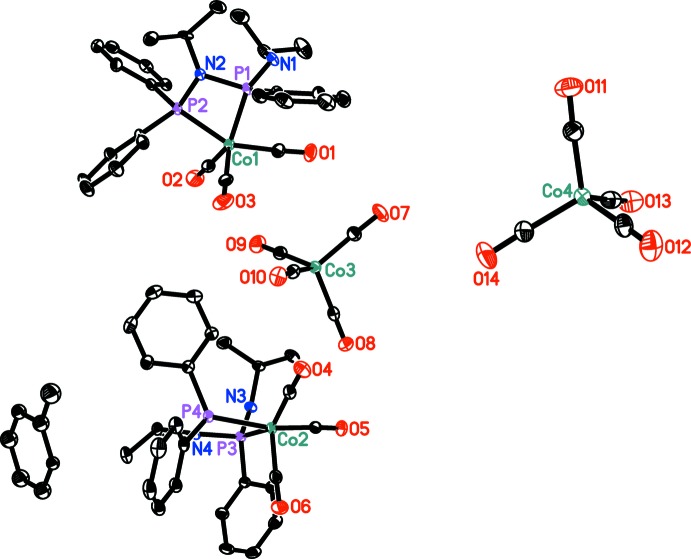
The asymmetric unit of the title compound. Hydrogen atoms are omitted for clarity. Displacement ellipsoids correspond to the 30% probability level. Only one orientation of the disordered toluene mol­ecule is shown.

**Figure 2:**
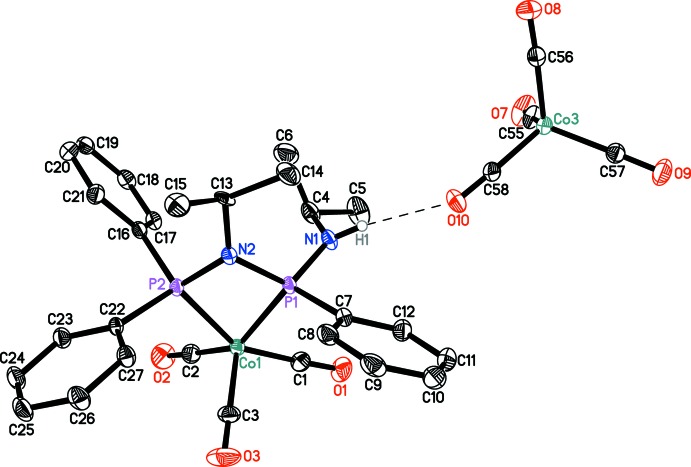
Inter­ionic N—H⋯O hydrogen bond (dashed line) connecting an ion-pair in the title compound. Hydrogen atoms not involved in hydrogen bonding, the co-crystallized toluene mol­ecule and the second ion-pair of the asymmetric unit are omitted for clarity. Displacement ellipsoids are drawn at the 30% probability level.

The title compound crystallizes in the monoclinic space group *P*2_1_/*n* with eight cations, eight anions as well as two mol­ecules of toluene in the unit cell. The toluene solvent mol­ecules are found to be disordered about inversion centres. The asymmetric unit is shown in Fig. 1 ▶. In the cation, the Co^I^ atom is fivefold coordinated by three carbonyl ligands and the PNPNH ligand, which is bound *via* both P atoms (Fig. 2 ▶). Thus, the bidentate ligand forms a four-membered metallacycle at the Co^I^ atom with the central N atom being tilted out of the plane formed by the Co and the two P atoms [the dihedral angles between the CoP_2_ and NP_2_ planes are 15.73 (10) and 14.44 (9)°]. The terminal secondary amine is not involved in complexation with the Co^I^ atom and acts as a spectator group. In the cyclic units the following bond lengths are observed: Co1—P1 2.1948 (7), P1—N2 1.698 (2), N2—P2 1.695 (2), P2—Co1 2.1800 (7), Co2—P3 2.1884 (7), P3—N4 1.695 (2), N4—P4 1.702 (2), P4—Co2 2.1971 (7) Å. A similar coordination mode was previously found for this ligand in a variety of transition metal complexes (Aluri *et al.*, 2010 ▶). In the cationic parts, one of the Co—C distances [Co1—C3 1.821 (3) and Co2—C30 1.832 (3) Å] is slightly longer than the other two values. In the anion, the geometry at the cobalt atom is found to be distorted tetra­hedral; all C—Co—C angles are found to be between 105.75 (13) and 111.89 (14)°, thus indicating a minor deviation from ideal *T_d_* symmetry. The Co—C bond lengths in the anions vary from 1.754 (4)–1.770 (3) Å and are comparable to those observed for a range of complexes displaying tetra­carbonyl­cobaltate anions (*vide supra*), including ionic salts of tetra­carbonyl­cobaltate with alkali (Klüfers, 1984*a* ▶,*b* ▶) and ammonium cations (Brammer *et al.* 1992 ▶; Brammer & Zhao, 1995 ▶).

## Supra­molecular features   

A weak hydrogen-bonding inter­action is observed between the NH group of the cation and one of the O atoms of the tetra­carbonyl­cobaltate(−I) anions (Table 1 ▶). Other than in the literature-known compound [Et_3_NH][Co(CO)_4_] (Brammer *et al.*, 1992 ▶), no 3*c*–4*e* hydrogen-bond-like N—H⋯Co inter­action has been found.

## Database survey   

For a similar, carbonyl-bridged dinuclear cobalt complex with this PNPNH ligand, see: Hansen *et al.* (2013 ▶). Examples for structural reports of other Co^I^–Co^−I^ ion-pair complexes can be found in Fellmann *et al.* (1983 ▶), Bockman & Kochi (1989 ▶), Zhang *et al.* (1994 ▶), Uehara *et al.* (2005 ▶), van Rensburg *et al.* (2007 ▶) and Azhakar *et al.* (2012 ▶). Other transition metal complexes with this ligand are described in Aluri *et al.* (2010 ▶) and Dulai *et al.* (2011 ▶).

## Synthesis and crystallization   

General: *N*-(diphenylphosphanyl)-*N*,*N′*-diisopropyl-di­amino­phenyl­phosphine was synthesized by a literature method (Peitz *et al.*, 2010 ▶). Co_2_(CO)_8_ was purchased from Strem and used without further purification. Toluene was dried over CaH_2_ and distilled prior to use. Synthesis: A solution of Co_2_(CO)_8_ (0.30 g, 0.88 mmol) in toluene (10 ml) was added to *N*-(diphenylphosphanyl)-*N*,*N′*-diisopropyl-di­amino­phenyl­phos­phine, Ph_2_PN(*i*-Pr)P(Ph)N(H)*i*-Pr (0.36 g, 0.88 mmol) in a glove box. After gas evolution subsided, the 50 ml Schlenk flask was closed and heated to 383 K for 35 min without stirring to preserve the two-phase system. After crystallization from toluene at room temperature for three days, two crystal fractions were separated from the solvent and washed with *n*-hexane (2 × 5 ml). The fraction of cubic brown crystals showed space-group and lattice parameters identical to X-ray diffraction data published previously (Hansen *et al.*, 2013 ▶). The second fraction contained yellow needles with the crystal structure presented here. Further isolation of this new complex was not possible as it inevitably forms the known dinuclear product when dissolved in organic solvents. Manual picking of the crystals was difficult as the material proved too delicate. Analytics: ^31^P NMR (297 K, THF-*d*_8_, 162 Hz): δ (p.p.m) 61.8 (*d*, *J* = 150 Hz), 59.9 (*d*, *J* = 150 Hz); IR (ATR, THF): ν^−1^ [cm^−1^] 3335 (*w*), 3191 (*w*), 3058 (*w*), 2975 (*m*), 2869 (*m*), 2081 [*s*, Co(CO)_3_^+^], 2021 [*s*, Co(CO)_3_^+^], 1979 (*w*), 1872 [*s*, Co(CO)_4_^−^], 1586 (*w*), 1462 (*w*), 1436 (*m*), 1390 (*w*), 1369 (*w*), 1311(*w*), 1165 (*w*), 1125 (*m*), 1097 (*m*), 1064 (*m*), 999 (*w*), 896 (*m*), 869 (*m*), 747 (*m*), 716 (*w*), 694 (*m*), 631 (*w*), 612 (*w*), 550 (*s*), 501 (*m*), 426 (*m*).

## Refinement   

Crystal data, data collection and structure refinement details are summarized in Table 2 ▶. Atoms H1 and H3 were located in a difference Fourier map and their coordinates were refined with the restraint N—H = 0.87 (1) Å. All other H atoms were placed in idealized positions with *d*(C—H) = 0.95–1.00 (CH) and 0.98 Å (CH_3_) and refined using a riding model with *U*_iso_(H) fixed at 1.2 *U*_eq_(C) for CH and 1.5 *U*_eq_(C) for CH_3_. The ring of the half-occupied toluene mol­ecule was constrained to resemble an ideal hexa­gon with C—C distances of 1.39 Å. SADI instructions were used to improve the geometry of one phenyl ring (C24–C25, C25–C26) and one *i*-propyl group (C13–C14, C13–C15).

## Supplementary Material

Crystal structure: contains datablock(s) I. DOI: 10.1107/S1600536814024908/rz5139sup1.cif

Structure factors: contains datablock(s) I. DOI: 10.1107/S1600536814024908/rz5139Isup2.hkl

CCDC reference: 1033917

Additional supporting information:  crystallographic information; 3D view; checkCIF report

## Figures and Tables

**Table 1:** Hydrogen-bond geometry (, )

*D*H*A*	*D*H	H*A*	*D* *A*	*D*H*A*
N1H1O10^i^	0.87(1)	2.22(2)	3.041(3)	159(3)
N3H3O13^ii^	0.86(1)	2.27(1)	3.101(3)	163(3)

Symmetry codes: (i) 
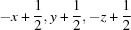
; (ii) 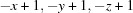.

**Table 2:** Experimental details

Crystal data	
Chemical formula	[Co(C_24_H_30_N_2_P_2_)(CO)_3_][Co(CO)_4_]0.25C_7_H_8_
*M*_r_	745.40
Crystal system, space group	Monoclinic, *P*2_1_/*n*
Temperature (K)	150
*a*, *b*, *c* ()	22.1602(6), 12.9730(3), 24.7883(6)
()	103.9330(12)
*V* (^3^)	6916.6(3)
*Z*	8
Radiation type	Cu *K*
(mm^1^)	8.79
Crystal size (mm)	0.43 0.11 0.05

Data collection	
Diffractometer	Bruker Kappa APEXII DUO
Absorption correction	Multi-scan (*SADABS*; Bruker, 2011 ▶)
*T*_min_, *T*_max_	0.12, 0.65
No. of measured, independent and observed [*I* > 2(*I*)] reflections	89837, 12171, 11080
*R*_int_	0.044
(sin /)_max_ (^1^)	0.595

Refinement	
*R*[*F* ^2^ > 2(*F* ^2^)], *wR*(*F* ^2^), *S*	0.038, 0.100, 1.03
No. of reflections	12171
No. of parameters	825
No. of restraints	4
H-atom treatment	H atoms treated by a mixture of independent and constrained refinement
_max_, _min_ (e ^3^)	0.93, 0.57

Computer programs: *APEX2* and *SAINT* (Bruker, 2011 ▶), *SHELXS97*, *SHELXL2014* and *SHELXTL* (Sheldrick, 2008 ▶).

## supplementary crystallographic information

### Crystal data

**Table:**

[Co(C_24_H_30_N_2_P_2_)(CO)_3_][Co(CO)_4_]·0.25C_7_H_8_	*F*(000) = 3060
*M**_r_* = 745.40	*D*_x_ = 1.432 Mg m^−^^3^
Monoclinic, *P*2_1_/*n*	Cu *K*α radiation, λ = 1.54178 Å
*a* = 22.1602 (6) Å	Cell parameters from 9777 reflections
*b* = 12.9730 (3) Å	θ = 3.7–66.7°
*c* = 24.7883 (6) Å	µ = 8.79 mm^−^^1^
β = 103.9330 (12)°	*T* = 150 K
*V* = 6916.6 (3) Å^3^	Needle, yellow
*Z* = 8	0.43 × 0.11 × 0.05 mm

### Data collection

**Table:**

Bruker Kappa APEXII DUO diffractometer	12171 independent reflections
Radiation source: microfocus	11080 reflections with *I* > 2σ(*I*)
Multilayer monochromator	*R*_int_ = 0.044
Detector resolution: 8.3333 pixels mm^-1^	θ_max_ = 66.5°, θ_min_ = 2.4°
φ and ω scans	*h* = −26→25
Absorption correction: multi-scan (*SADABS*; Bruker, 2011)	*k* = −15→14
*T*_min_ = 0.12, *T*_max_ = 0.65	*l* = −23→29
89837 measured reflections	

### Refinement

**Table:**

Refinement on *F*^2^	4 restraints
Least-squares matrix: full	Hydrogen site location: mixed
*R*[*F*^2^ > 2σ(*F*^2^)] = 0.038	H atoms treated by a mixture of independent and constrained refinement
*wR*(*F*^2^) = 0.100	*w* = 1/[σ^2^(*F*_o_^2^) + (0.0494*P*)^2^ + 7.7784*P*] where *P* = (*F*_o_^2^ + 2*F*_c_^2^)/3
*S* = 1.03	(Δ/σ)_max_ = 0.001
12171 reflections	Δρ_max_ = 0.93 e Å^−^^3^
825 parameters	Δρ_min_ = −0.57 e Å^−^^3^

### Special details

**Table:**

Geometry. All e.s.d.'s (except the e.s.d. in the dihedral angle between two l.s. planes) are estimated using the full covariance matrix. The cell e.s.d.'s are taken into account individually in the estimation of e.s.d.'s in distances, angles and torsion angles; correlations between e.s.d.'s in cell parameters are only used when they are defined by crystal symmetry. An approximate (isotropic) treatment of cell e.s.d.'s is used for estimating e.s.d.'s involving l.s. planes.

### Fractional atomic coordinates and isotropic or equivalent isotropic displacement parameters (Å^2^)

**Table:**

	*x*	*y*	*z*	*U*_iso_*/*U*_eq_	Occ. (<1)
C1	0.26645 (13)	0.8434 (2)	0.15492 (12)	0.0349 (6)	
C2	0.25541 (13)	0.7575 (2)	0.05615 (13)	0.0368 (7)	
C3	0.25514 (13)	0.6280 (2)	0.15325 (14)	0.0384 (7)	
C4	0.12520 (16)	0.9773 (2)	0.10407 (12)	0.0387 (7)	
H4	0.1533	0.9488	0.0816	0.046*	
C5	0.1505 (2)	1.0822 (3)	0.12626 (17)	0.0603 (11)	
H5A	0.1226	1.1132	0.1470	0.090*	
H5B	0.1531	1.1271	0.0951	0.090*	
H5C	0.1919	1.0737	0.1510	0.090*	
C6	0.06036 (18)	0.9842 (3)	0.06670 (15)	0.0571 (10)	
H6A	0.0472	0.9159	0.0514	0.086*	
H6B	0.0603	1.0324	0.0363	0.086*	
H6C	0.0315	1.0087	0.0883	0.086*	
C7	0.15272 (13)	0.7513 (2)	0.22363 (11)	0.0292 (6)	
C8	0.13489 (14)	0.6542 (2)	0.23867 (12)	0.0348 (6)	
H8	0.1163	0.6062	0.2107	0.042*	
C9	0.14415 (16)	0.6277 (3)	0.29406 (13)	0.0446 (8)	
H9	0.1315	0.5619	0.3040	0.053*	
C10	0.17175 (17)	0.6966 (3)	0.33486 (13)	0.0503 (9)	
H10	0.1781	0.6782	0.3729	0.060*	
C11	0.19017 (17)	0.7918 (3)	0.32061 (13)	0.0480 (8)	
H11	0.2094	0.8390	0.3489	0.058*	
C12	0.18085 (14)	0.8195 (2)	0.26511 (12)	0.0374 (7)	
H12	0.1938	0.8854	0.2555	0.045*	
C13	0.02566 (11)	0.70193 (17)	0.10262 (9)	0.0246 (5)	
H13	0.0072	0.7400	0.0674	0.029*	
C14	0.00085 (14)	0.7512 (3)	0.14945 (13)	0.0432 (8)	
H14A	0.0192	0.7164	0.1847	0.065*	
H14B	−0.0445	0.7442	0.1409	0.065*	
H14C	0.0120	0.8244	0.1526	0.065*	
C15	0.00114 (14)	0.5900 (2)	0.09563 (15)	0.0422 (7)	
H15A	0.0162	0.5560	0.0661	0.063*	
H15B	−0.0444	0.5906	0.0858	0.063*	
H15C	0.0161	0.5524	0.1306	0.063*	
C16	0.09747 (12)	0.72904 (19)	−0.00255 (10)	0.0247 (5)	
C17	0.11839 (13)	0.8191 (2)	−0.02320 (11)	0.0283 (6)	
H17	0.1535	0.8546	−0.0017	0.034*	
C18	0.08784 (14)	0.8567 (2)	−0.07507 (11)	0.0337 (6)	
H18	0.1026	0.9173	−0.0891	0.040*	
C19	0.03626 (14)	0.8065 (2)	−0.10626 (12)	0.0368 (7)	
H19	0.0155	0.8326	−0.1417	0.044*	
C20	0.01487 (14)	0.7183 (3)	−0.08597 (12)	0.0392 (7)	
H20	−0.0209	0.6842	−0.1073	0.047*	
C21	0.04531 (13)	0.6793 (2)	−0.03476 (11)	0.0329 (6)	
H21	0.0306	0.6180	−0.0213	0.039*	
C22	0.14085 (12)	0.54130 (19)	0.05436 (10)	0.0237 (5)	
C23	0.15779 (14)	0.5072 (2)	0.00688 (11)	0.0329 (6)	
H23	0.1593	0.5542	−0.0221	0.039*	
C24	0.17251 (16)	0.4042 (2)	0.00199 (12)	0.0411 (7)	
H24	0.1841	0.3808	−0.0304	0.049*	
C25	0.17039 (15)	0.3355 (2)	0.04412 (10)	0.0421 (7)	
H25	0.1805	0.2651	0.0407	0.050*	
C26	0.15350 (15)	0.3695 (2)	0.09130 (12)	0.0379 (7)	
H26	0.1517	0.3220	0.1200	0.046*	
C27	0.13923 (13)	0.4717 (2)	0.09699 (11)	0.0299 (6)	
H27	0.1283	0.4947	0.1298	0.036*	
C28	0.58062 (13)	0.4567 (2)	0.27082 (11)	0.0308 (6)	
C29	0.67739 (12)	0.5553 (2)	0.25305 (10)	0.0277 (6)	
C30	0.69210 (14)	0.3421 (2)	0.26735 (11)	0.0335 (6)	
C31	0.58671 (12)	0.6413 (2)	0.12397 (11)	0.0259 (5)	
H31	0.5690	0.6143	0.1547	0.031*	
C32	0.53701 (14)	0.6327 (3)	0.06988 (12)	0.0399 (7)	
H32A	0.5535	0.6585	0.0392	0.060*	
H32B	0.5007	0.6737	0.0725	0.060*	
H32C	0.5248	0.5604	0.0631	0.060*	
C33	0.60641 (15)	0.7521 (2)	0.13732 (13)	0.0380 (7)	
H33A	0.6385	0.7545	0.1723	0.057*	
H33B	0.5704	0.7928	0.1410	0.057*	
H33C	0.6231	0.7806	0.1073	0.057*	
C34	0.73787 (11)	0.4484 (2)	0.14454 (10)	0.0238 (5)	
C35	0.76054 (13)	0.3498 (2)	0.13827 (11)	0.0320 (6)	
H35	0.7337	0.2918	0.1348	0.038*	
C36	0.82267 (14)	0.3365 (3)	0.13713 (12)	0.0399 (7)	
H36	0.8381	0.2694	0.1328	0.048*	
C37	0.86170 (13)	0.4201 (3)	0.14227 (12)	0.0406 (7)	
H37	0.9039	0.4107	0.1412	0.049*	
C38	0.83978 (13)	0.5175 (3)	0.14893 (12)	0.0382 (7)	
H38	0.8671	0.5750	0.1526	0.046*	
C39	0.77817 (13)	0.5323 (2)	0.15025 (11)	0.0296 (6)	
H39	0.7634	0.5996	0.1550	0.036*	
C40	0.59574 (12)	0.3382 (2)	0.05627 (10)	0.0267 (5)	
H40	0.5505	0.3551	0.0435	0.032*	
C41	0.60339 (16)	0.2242 (2)	0.04650 (12)	0.0372 (7)	
H41A	0.6474	0.2055	0.0587	0.056*	
H41B	0.5883	0.2089	0.0068	0.056*	
H41C	0.5794	0.1843	0.0677	0.056*	
C42	0.63040 (15)	0.4023 (2)	0.02205 (11)	0.0345 (6)	
H42A	0.6215	0.4755	0.0260	0.052*	
H42B	0.6168	0.3824	−0.0172	0.052*	
H42C	0.6752	0.3902	0.0352	0.052*	
C43	0.58053 (13)	0.2016 (2)	0.18317 (10)	0.0276 (6)	
C44	0.53245 (15)	0.1575 (2)	0.20273 (12)	0.0364 (7)	
H44	0.4973	0.1977	0.2051	0.044*	
C45	0.53624 (17)	0.0543 (3)	0.21875 (13)	0.0458 (8)	
H45	0.5037	0.0240	0.2322	0.055*	
C46	0.58709 (18)	−0.0042 (2)	0.21511 (12)	0.0458 (8)	
H46	0.5892	−0.0747	0.2257	0.055*	
C47	0.63477 (16)	0.0394 (2)	0.19621 (12)	0.0405 (7)	
H47	0.6697	−0.0013	0.1938	0.049*	
C48	0.63226 (14)	0.1421 (2)	0.18073 (11)	0.0332 (6)	
H48	0.6657	0.1720	0.1685	0.040*	
C49	0.49608 (12)	0.3638 (2)	0.13700 (10)	0.0263 (5)	
C50	0.46769 (12)	0.4538 (2)	0.14867 (11)	0.0296 (6)	
H50	0.4899	0.5005	0.1758	0.036*	
C51	0.40676 (13)	0.4755 (2)	0.12067 (12)	0.0367 (7)	
H51	0.3875	0.5371	0.1286	0.044*	
C52	0.37425 (14)	0.4077 (3)	0.08138 (13)	0.0404 (7)	
H52	0.3327	0.4230	0.0623	0.049*	
C53	0.40198 (14)	0.3178 (3)	0.06969 (12)	0.0397 (7)	
H53	0.3794	0.2716	0.0425	0.048*	
C54	0.46254 (13)	0.2946 (2)	0.09748 (11)	0.0331 (6)	
H54	0.4812	0.2322	0.0898	0.040*	
C55	0.42419 (15)	0.8392 (3)	0.26588 (12)	0.0401 (7)	
C56	0.52940 (14)	0.7284 (2)	0.24900 (11)	0.0316 (6)	
C57	0.42198 (14)	0.7594 (2)	0.15653 (12)	0.0349 (6)	
C58	0.41993 (13)	0.6173 (2)	0.24610 (11)	0.0338 (6)	
C59	0.14719 (16)	0.1170 (3)	0.95283 (15)	0.0474 (8)	
C60	0.25789 (16)	0.0432 (2)	1.02628 (14)	0.0439 (8)	
C61	0.26473 (15)	0.2103 (3)	0.95163 (13)	0.0412 (7)	
C62	0.23996 (18)	0.0006 (3)	0.91221 (15)	0.0587 (10)	
Co1	0.22216 (2)	0.74279 (3)	0.11504 (2)	0.02425 (10)	
Co2	0.63667 (2)	0.43778 (3)	0.23007 (2)	0.02064 (10)	
Co3	0.44772 (2)	0.73693 (3)	0.22886 (2)	0.02648 (11)	
Co4	0.22726 (2)	0.09426 (4)	0.95973 (2)	0.03635 (12)	
N1	0.12681 (11)	0.90667 (17)	0.15071 (10)	0.0306 (5)	
N2	0.09256 (10)	0.70471 (16)	0.11124 (9)	0.0243 (4)	
N3	0.64182 (10)	0.57857 (16)	0.12197 (9)	0.0234 (4)	
N4	0.61636 (10)	0.36438 (16)	0.11707 (8)	0.0218 (4)	
O1	0.29238 (11)	0.90745 (18)	0.18266 (10)	0.0517 (6)	
O2	0.27494 (12)	0.7675 (2)	0.01802 (11)	0.0610 (7)	
O3	0.27388 (12)	0.5557 (2)	0.17721 (13)	0.0676 (8)	
O4	0.54660 (11)	0.46924 (18)	0.29796 (9)	0.0458 (5)	
O5	0.70530 (9)	0.62862 (16)	0.26537 (8)	0.0380 (5)	
O6	0.72663 (12)	0.28503 (19)	0.29185 (10)	0.0553 (6)	
O7	0.41050 (14)	0.9060 (2)	0.29101 (10)	0.0648 (8)	
O8	0.58259 (10)	0.72001 (18)	0.26369 (9)	0.0455 (5)	
O9	0.40486 (12)	0.7722 (2)	0.10943 (9)	0.0554 (6)	
O10	0.40428 (11)	0.53707 (18)	0.25765 (10)	0.0492 (6)	
O11	0.09514 (12)	0.1332 (2)	0.95002 (13)	0.0701 (8)	
O12	0.27831 (15)	0.0082 (2)	1.06974 (11)	0.0703 (8)	
O13	0.28943 (12)	0.2881 (2)	0.94823 (11)	0.0555 (6)	
O14	0.24920 (18)	−0.0636 (3)	0.88265 (13)	0.0971 (12)	
P1	0.14487 (3)	0.78514 (5)	0.15180 (3)	0.02196 (13)	
P2	0.13585 (3)	0.67810 (5)	0.06516 (2)	0.02233 (14)	
P3	0.65869 (3)	0.46497 (5)	0.14979 (2)	0.01982 (13)	
P4	0.57780 (3)	0.33814 (5)	0.16712 (2)	0.02187 (13)	
H1	0.1107 (15)	0.931 (3)	0.1765 (11)	0.046 (10)*	
H3	0.6629 (13)	0.603 (2)	0.0999 (11)	0.040 (9)*	
C63	0.46917 (16)	0.0090 (4)	0.02383 (17)	0.0476 (16)*	0.5
C64	0.52811 (19)	−0.0277 (3)	0.04918 (13)	0.0308 (12)*	0.5
H64	0.5381	−0.0448	0.0876	0.037*	0.5
C65	0.57243 (14)	−0.0394 (4)	0.0184 (2)	0.0521 (17)*	0.5
H65	0.6127	−0.0644	0.0357	0.062*	0.5
C66	0.55781 (19)	−0.0143 (4)	−0.03781 (19)	0.0560 (19)*	0.5
H66	0.5881	−0.0223	−0.0589	0.067*	0.5
C67	0.4989 (2)	0.0223 (4)	−0.06317 (13)	0.0567 (18)*	0.5
H67	0.4889	0.0394	−0.1016	0.068*	0.5
C68	0.45455 (15)	0.0340 (4)	−0.03235 (17)	0.0410 (14)*	0.5
H68	0.4143	0.0591	−0.0497	0.049*	0.5
C69	0.4213 (4)	0.0175 (8)	0.0563 (4)	0.073 (2)*	0.5
H69A	0.3827	0.0444	0.0325	0.110*	0.5
H69B	0.4136	−0.0507	0.0703	0.110*	0.5
H69C	0.4361	0.0644	0.0877	0.110*	0.5

### Atomic displacement parameters (Å^2^)

**Table:**

	*U*^11^	*U*^22^	*U*^33^	*U*^12^	*U*^13^	*U*^23^
C1	0.0331 (15)	0.0352 (16)	0.0358 (15)	−0.0044 (13)	0.0071 (12)	−0.0001 (13)
C2	0.0273 (14)	0.0455 (18)	0.0386 (16)	−0.0011 (13)	0.0099 (13)	−0.0077 (14)
C3	0.0261 (14)	0.0319 (16)	0.0515 (18)	0.0030 (12)	−0.0016 (13)	−0.0017 (14)
C4	0.0578 (19)	0.0275 (15)	0.0358 (15)	0.0094 (14)	0.0211 (14)	0.0036 (12)
C5	0.103 (3)	0.0238 (17)	0.064 (2)	−0.0030 (18)	0.040 (2)	0.0042 (16)
C6	0.062 (2)	0.063 (2)	0.0479 (19)	0.0244 (19)	0.0146 (17)	0.0098 (18)
C7	0.0313 (14)	0.0300 (15)	0.0259 (13)	0.0053 (11)	0.0062 (11)	−0.0057 (11)
C8	0.0430 (16)	0.0257 (15)	0.0365 (15)	0.0071 (12)	0.0114 (13)	0.0033 (12)
C9	0.059 (2)	0.0366 (17)	0.0432 (18)	0.0176 (15)	0.0215 (15)	0.0124 (14)
C10	0.068 (2)	0.054 (2)	0.0285 (16)	0.0254 (18)	0.0115 (15)	0.0094 (15)
C11	0.060 (2)	0.052 (2)	0.0280 (15)	0.0149 (17)	0.0038 (14)	−0.0061 (14)
C12	0.0458 (17)	0.0331 (16)	0.0315 (15)	0.0026 (13)	0.0056 (13)	−0.0034 (12)
C13	0.0241 (12)	0.0321 (14)	0.0225 (12)	0.0157 (11)	0.0152 (10)	0.0105 (11)
C14	0.0346 (16)	0.0487 (19)	0.0525 (19)	0.0037 (14)	0.0228 (14)	−0.0057 (15)
C15	0.0276 (15)	0.0387 (17)	0.061 (2)	−0.0103 (13)	0.0125 (14)	−0.0064 (15)
C16	0.0299 (13)	0.0203 (13)	0.0244 (13)	0.0029 (10)	0.0074 (10)	−0.0026 (10)
C17	0.0325 (14)	0.0262 (14)	0.0280 (13)	0.0012 (11)	0.0107 (11)	−0.0032 (11)
C18	0.0467 (17)	0.0284 (15)	0.0294 (14)	0.0079 (12)	0.0156 (13)	0.0052 (12)
C19	0.0421 (16)	0.0413 (17)	0.0254 (14)	0.0169 (14)	0.0051 (12)	−0.0010 (13)
C20	0.0347 (15)	0.0440 (18)	0.0341 (15)	0.0054 (13)	−0.0013 (12)	−0.0081 (13)
C21	0.0358 (15)	0.0302 (15)	0.0305 (14)	−0.0014 (12)	0.0038 (12)	−0.0034 (12)
C22	0.0263 (12)	0.0168 (12)	0.0266 (13)	−0.0023 (10)	0.0035 (10)	−0.0057 (10)
C23	0.0431 (16)	0.0297 (15)	0.0260 (13)	0.0050 (12)	0.0086 (12)	−0.0016 (11)
C24	0.058 (2)	0.0323 (16)	0.0341 (16)	0.0108 (14)	0.0126 (14)	−0.0070 (13)
C25	0.0562 (19)	0.0223 (15)	0.0483 (18)	0.0065 (13)	0.0137 (15)	−0.0055 (13)
C26	0.0497 (18)	0.0220 (14)	0.0435 (17)	0.0017 (13)	0.0141 (14)	0.0037 (12)
C27	0.0370 (15)	0.0225 (14)	0.0316 (14)	−0.0025 (11)	0.0111 (11)	−0.0017 (11)
C28	0.0402 (16)	0.0252 (14)	0.0274 (13)	−0.0006 (12)	0.0088 (12)	0.0016 (11)
C29	0.0309 (14)	0.0301 (15)	0.0217 (12)	0.0052 (12)	0.0057 (10)	0.0010 (11)
C30	0.0425 (16)	0.0292 (15)	0.0267 (14)	0.0040 (13)	0.0045 (12)	−0.0010 (12)
C31	0.0271 (13)	0.0229 (13)	0.0284 (13)	0.0072 (10)	0.0085 (10)	0.0048 (11)
C32	0.0358 (16)	0.0500 (19)	0.0314 (15)	0.0119 (14)	0.0035 (12)	0.0052 (14)
C33	0.0496 (18)	0.0221 (14)	0.0458 (17)	0.0091 (13)	0.0182 (14)	0.0039 (13)
C34	0.0259 (13)	0.0280 (14)	0.0174 (11)	0.0044 (10)	0.0050 (10)	0.0019 (10)
C35	0.0336 (14)	0.0312 (15)	0.0309 (14)	0.0070 (12)	0.0074 (11)	0.0018 (12)
C36	0.0386 (16)	0.0489 (19)	0.0332 (15)	0.0211 (15)	0.0102 (12)	0.0004 (14)
C37	0.0260 (14)	0.065 (2)	0.0319 (15)	0.0107 (14)	0.0091 (12)	0.0035 (14)
C38	0.0265 (14)	0.055 (2)	0.0332 (15)	−0.0051 (13)	0.0074 (11)	0.0022 (14)
C39	0.0317 (14)	0.0311 (15)	0.0262 (13)	0.0006 (11)	0.0073 (11)	0.0001 (11)
C40	0.0323 (14)	0.0279 (14)	0.0193 (12)	−0.0003 (11)	0.0047 (10)	−0.0017 (10)
C41	0.0565 (19)	0.0288 (15)	0.0279 (14)	−0.0056 (13)	0.0134 (13)	−0.0059 (12)
C42	0.0522 (18)	0.0325 (16)	0.0209 (13)	−0.0021 (13)	0.0126 (12)	0.0000 (11)
C43	0.0389 (15)	0.0214 (13)	0.0212 (12)	−0.0057 (11)	0.0046 (11)	0.0000 (10)
C44	0.0478 (17)	0.0311 (16)	0.0316 (14)	−0.0071 (13)	0.0121 (13)	0.0026 (12)
C45	0.065 (2)	0.0358 (18)	0.0384 (17)	−0.0171 (16)	0.0149 (15)	0.0074 (14)
C46	0.080 (2)	0.0210 (15)	0.0310 (15)	−0.0076 (16)	0.0035 (15)	0.0036 (12)
C47	0.060 (2)	0.0227 (15)	0.0348 (15)	0.0058 (14)	0.0037 (14)	0.0009 (12)
C48	0.0438 (16)	0.0224 (14)	0.0315 (14)	−0.0005 (12)	0.0052 (12)	0.0007 (11)
C49	0.0276 (13)	0.0278 (14)	0.0244 (12)	−0.0022 (11)	0.0083 (10)	0.0028 (11)
C50	0.0294 (14)	0.0310 (15)	0.0299 (14)	−0.0017 (11)	0.0098 (11)	0.0022 (12)
C51	0.0325 (15)	0.0393 (17)	0.0399 (16)	0.0047 (13)	0.0120 (12)	0.0091 (13)
C52	0.0286 (14)	0.054 (2)	0.0374 (16)	0.0003 (14)	0.0063 (12)	0.0116 (14)
C53	0.0371 (16)	0.0511 (19)	0.0285 (14)	−0.0126 (14)	0.0034 (12)	0.0005 (14)
C54	0.0337 (15)	0.0361 (16)	0.0295 (14)	−0.0038 (12)	0.0074 (11)	−0.0001 (12)
C55	0.0498 (18)	0.0395 (18)	0.0292 (15)	0.0122 (14)	0.0060 (13)	0.0030 (14)
C56	0.0402 (17)	0.0268 (14)	0.0284 (14)	−0.0024 (12)	0.0094 (12)	−0.0023 (11)
C57	0.0342 (15)	0.0372 (16)	0.0335 (16)	0.0019 (12)	0.0084 (12)	0.0022 (13)
C58	0.0326 (14)	0.0422 (18)	0.0272 (14)	−0.0020 (13)	0.0085 (11)	0.0028 (13)
C59	0.046 (2)	0.0408 (19)	0.054 (2)	0.0012 (15)	0.0078 (15)	−0.0026 (15)
C60	0.057 (2)	0.0323 (17)	0.0434 (19)	−0.0089 (15)	0.0130 (15)	−0.0044 (14)
C61	0.0395 (16)	0.052 (2)	0.0347 (16)	0.0096 (15)	0.0146 (13)	0.0109 (15)
C62	0.058 (2)	0.072 (3)	0.0418 (19)	0.015 (2)	0.0039 (16)	−0.0135 (19)
Co1	0.0222 (2)	0.0224 (2)	0.0275 (2)	−0.00050 (16)	0.00468 (17)	−0.00343 (17)
Co2	0.0264 (2)	0.0172 (2)	0.01802 (19)	0.00094 (16)	0.00476 (15)	0.00057 (15)
Co3	0.0298 (2)	0.0261 (2)	0.0237 (2)	0.00174 (17)	0.00678 (17)	0.00064 (17)
Co4	0.0363 (3)	0.0368 (3)	0.0357 (3)	0.0031 (2)	0.0083 (2)	−0.0046 (2)
N1	0.0434 (13)	0.0199 (12)	0.0313 (12)	0.0052 (10)	0.0147 (10)	−0.0033 (9)
N2	0.0260 (11)	0.0217 (11)	0.0251 (11)	0.0002 (9)	0.0061 (9)	−0.0036 (9)
N3	0.0268 (11)	0.0207 (11)	0.0249 (11)	0.0044 (9)	0.0105 (9)	0.0044 (9)
N4	0.0285 (11)	0.0184 (10)	0.0191 (10)	−0.0001 (8)	0.0067 (8)	−0.0001 (8)
O1	0.0507 (14)	0.0445 (14)	0.0541 (14)	−0.0169 (11)	0.0014 (11)	−0.0130 (11)
O2	0.0473 (14)	0.094 (2)	0.0504 (14)	−0.0018 (13)	0.0283 (12)	−0.0065 (14)
O3	0.0497 (14)	0.0401 (14)	0.097 (2)	0.0078 (12)	−0.0137 (14)	0.0197 (14)
O4	0.0544 (13)	0.0510 (14)	0.0409 (12)	0.0026 (11)	0.0290 (11)	0.0010 (10)
O5	0.0402 (11)	0.0326 (11)	0.0392 (11)	−0.0102 (9)	0.0055 (9)	−0.0072 (9)
O6	0.0651 (16)	0.0451 (14)	0.0465 (13)	0.0258 (12)	−0.0048 (12)	0.0053 (11)
O7	0.093 (2)	0.0541 (16)	0.0451 (14)	0.0320 (15)	0.0130 (13)	−0.0114 (12)
O8	0.0317 (12)	0.0534 (14)	0.0494 (13)	−0.0013 (10)	0.0058 (10)	−0.0061 (11)
O9	0.0607 (15)	0.0723 (18)	0.0299 (12)	0.0053 (13)	0.0044 (11)	0.0079 (11)
O10	0.0568 (14)	0.0454 (14)	0.0480 (13)	−0.0147 (11)	0.0176 (11)	0.0109 (11)
O11	0.0411 (15)	0.0700 (19)	0.097 (2)	0.0080 (13)	0.0119 (14)	0.0024 (16)
O12	0.105 (2)	0.0587 (17)	0.0426 (15)	−0.0054 (16)	0.0096 (14)	0.0110 (13)
O13	0.0536 (14)	0.0556 (16)	0.0618 (16)	−0.0015 (12)	0.0226 (12)	0.0213 (13)
O14	0.112 (3)	0.112 (3)	0.0626 (19)	0.035 (2)	0.0114 (18)	−0.043 (2)
P1	0.0280 (3)	0.0161 (3)	0.0218 (3)	−0.0006 (2)	0.0061 (2)	−0.0033 (2)
P2	0.0286 (3)	0.0172 (3)	0.0208 (3)	−0.0005 (2)	0.0051 (2)	−0.0027 (2)
P3	0.0231 (3)	0.0169 (3)	0.0196 (3)	0.0016 (2)	0.0055 (2)	0.0007 (2)
P4	0.0270 (3)	0.0187 (3)	0.0202 (3)	−0.0010 (2)	0.0062 (2)	0.0004 (2)

### Geometric parameters (Å, º)

**Table:**

C1—O1	1.142 (4)	C37—C38	1.378 (5)
C1—Co1	1.782 (3)	C37—H37	0.9500
C2—O2	1.138 (4)	C38—C39	1.387 (4)
C2—Co1	1.797 (3)	C38—H38	0.9500
C3—O3	1.134 (4)	C39—H39	0.9500
C3—Co1	1.821 (3)	C40—N4	1.505 (3)
C4—N1	1.469 (4)	C40—C41	1.515 (4)
C4—C6	1.513 (5)	C40—C42	1.521 (4)
C4—C5	1.523 (5)	C40—H40	1.0000
C4—H4	1.0000	C41—H41A	0.9800
C5—H5A	0.9800	C41—H41B	0.9800
C5—H5B	0.9800	C41—H41C	0.9800
C5—H5C	0.9800	C42—H42A	0.9800
C6—H6A	0.9800	C42—H42B	0.9800
C6—H6B	0.9800	C42—H42C	0.9800
C6—H6C	0.9800	C43—C44	1.395 (4)
C7—C12	1.386 (4)	C43—C48	1.396 (4)
C7—C8	1.397 (4)	C43—P4	1.813 (3)
C7—P1	1.801 (3)	C44—C45	1.394 (4)
C8—C9	1.382 (4)	C44—H44	0.9500
C8—H8	0.9500	C45—C46	1.379 (5)
C9—C10	1.377 (5)	C45—H45	0.9500
C9—H9	0.9500	C46—C47	1.376 (5)
C10—C11	1.374 (5)	C46—H46	0.9500
C10—H10	0.9500	C47—C48	1.384 (4)
C11—C12	1.388 (4)	C47—H47	0.9500
C11—H11	0.9500	C48—H48	0.9500
C12—H12	0.9500	C49—C50	1.389 (4)
C13—N2	1.446 (3)	C49—C54	1.402 (4)
C13—C14	1.538 (3)	C49—P4	1.816 (3)
C13—C15	1.546 (3)	C50—C51	1.390 (4)
C13—H13	1.0000	C50—H50	0.9500
C14—H14A	0.9800	C51—C52	1.379 (5)
C14—H14B	0.9800	C51—H51	0.9500
C14—H14C	0.9800	C52—C53	1.382 (5)
C15—H15A	0.9800	C52—H52	0.9500
C15—H15B	0.9800	C53—C54	1.386 (4)
C15—H15C	0.9800	C53—H53	0.9500
C16—C21	1.395 (4)	C54—H54	0.9500
C16—C17	1.398 (4)	C55—O7	1.149 (4)
C16—P2	1.814 (3)	C55—Co3	1.762 (3)
C17—C18	1.389 (4)	C56—O8	1.152 (4)
C17—H17	0.9500	C56—Co3	1.761 (3)
C18—C19	1.379 (4)	C57—O9	1.149 (4)
C18—H18	0.9500	C57—Co3	1.770 (3)
C19—C20	1.379 (5)	C58—O10	1.155 (4)
C19—H19	0.9500	C58—Co3	1.760 (3)
C20—C21	1.382 (4)	C59—O11	1.158 (4)
C20—H20	0.9500	C59—Co4	1.766 (4)
C21—H21	0.9500	C60—O12	1.156 (4)
C22—C23	1.391 (4)	C60—Co4	1.755 (3)
C22—C27	1.397 (4)	C61—O13	1.161 (4)
C22—P2	1.802 (3)	C61—Co4	1.754 (4)
C23—C24	1.387 (4)	C62—O14	1.159 (5)
C23—H23	0.9500	C62—Co4	1.761 (4)
C24—C25	1.382 (3)	Co1—P2	2.1800 (7)
C24—H24	0.9500	Co1—P1	2.1948 (7)
C25—C26	1.383 (3)	Co2—P3	2.1884 (7)
C25—H25	0.9500	Co2—P4	2.1971 (7)
C26—C27	1.377 (4)	N1—P1	1.625 (2)
C26—H26	0.9500	N1—H1	0.865 (10)
C27—H27	0.9500	N2—P2	1.695 (2)
C28—O4	1.137 (3)	N2—P1	1.698 (2)
C28—Co2	1.797 (3)	N3—P3	1.632 (2)
C29—O5	1.136 (3)	N3—H3	0.862 (10)
C29—Co2	1.793 (3)	N4—P3	1.695 (2)
C30—O6	1.131 (4)	N4—P4	1.702 (2)
C30—Co2	1.832 (3)	P1—P2	2.5246 (8)
C31—N3	1.478 (3)	P3—P4	2.5445 (9)
C31—C33	1.515 (4)	C63—C64	1.3900
C31—C32	1.521 (4)	C63—C68	1.3900
C31—H31	1.0000	C63—C69	1.482 (10)
C32—H32A	0.9800	C64—C65	1.3900
C32—H32B	0.9800	C64—H64	0.9500
C32—H32C	0.9800	C65—C66	1.3900
C33—H33A	0.9800	C65—H65	0.9500
C33—H33B	0.9800	C66—C67	1.3900
C33—H33C	0.9800	C66—H66	0.9500
C34—C39	1.394 (4)	C67—C68	1.3900
C34—C35	1.396 (4)	C67—H67	0.9500
C34—P3	1.804 (3)	C68—H68	0.9500
C35—C36	1.394 (4)	C69—H69A	0.9800
C35—H35	0.9500	C69—H69B	0.9800
C36—C37	1.374 (5)	C69—H69C	0.9800
C36—H36	0.9500		
			
O1—C1—Co1	175.7 (3)	C44—C43—C48	119.5 (3)
O2—C2—Co1	178.2 (3)	C44—C43—P4	119.3 (2)
O3—C3—Co1	177.9 (3)	C48—C43—P4	121.0 (2)
N1—C4—C6	111.0 (3)	C45—C44—C43	119.6 (3)
N1—C4—C5	109.6 (3)	C45—C44—H44	120.2
C6—C4—C5	112.4 (3)	C43—C44—H44	120.2
N1—C4—H4	107.9	C46—C45—C44	120.2 (3)
C6—C4—H4	107.9	C46—C45—H45	119.9
C5—C4—H4	107.9	C44—C45—H45	119.9
C4—C5—H5A	109.5	C47—C46—C45	120.2 (3)
C4—C5—H5B	109.5	C47—C46—H46	119.9
H5A—C5—H5B	109.5	C45—C46—H46	119.9
C4—C5—H5C	109.5	C46—C47—C48	120.5 (3)
H5A—C5—H5C	109.5	C46—C47—H47	119.8
H5B—C5—H5C	109.5	C48—C47—H47	119.8
C4—C6—H6A	109.5	C47—C48—C43	120.0 (3)
C4—C6—H6B	109.5	C47—C48—H48	120.0
H6A—C6—H6B	109.5	C43—C48—H48	120.0
C4—C6—H6C	109.5	C50—C49—C54	119.5 (3)
H6A—C6—H6C	109.5	C50—C49—P4	121.7 (2)
H6B—C6—H6C	109.5	C54—C49—P4	118.6 (2)
C12—C7—C8	118.9 (3)	C49—C50—C51	120.0 (3)
C12—C7—P1	119.8 (2)	C49—C50—H50	120.0
C8—C7—P1	121.2 (2)	C51—C50—H50	120.0
C9—C8—C7	120.4 (3)	C52—C51—C50	120.2 (3)
C9—C8—H8	119.8	C52—C51—H51	119.9
C7—C8—H8	119.8	C50—C51—H51	119.9
C10—C9—C8	120.1 (3)	C51—C52—C53	120.2 (3)
C10—C9—H9	120.0	C51—C52—H52	119.9
C8—C9—H9	120.0	C53—C52—H52	119.9
C11—C10—C9	120.1 (3)	C52—C53—C54	120.3 (3)
C11—C10—H10	119.9	C52—C53—H53	119.8
C9—C10—H10	119.9	C54—C53—H53	119.8
C10—C11—C12	120.3 (3)	C53—C54—C49	119.7 (3)
C10—C11—H11	119.8	C53—C54—H54	120.1
C12—C11—H11	119.8	C49—C54—H54	120.1
C7—C12—C11	120.2 (3)	O7—C55—Co3	178.1 (3)
C7—C12—H12	119.9	O8—C56—Co3	177.4 (3)
C11—C12—H12	119.9	O9—C57—Co3	178.7 (3)
N2—C13—C14	114.7 (2)	O10—C58—Co3	177.0 (3)
N2—C13—C15	111.11 (19)	O11—C59—Co4	177.8 (3)
C14—C13—C15	107.2 (2)	O12—C60—Co4	178.9 (3)
N2—C13—H13	107.9	O13—C61—Co4	177.5 (3)
C14—C13—H13	107.9	O14—C62—Co4	177.3 (4)
C15—C13—H13	107.9	C1—Co1—C2	95.41 (14)
C13—C14—H14A	109.5	C1—Co1—C3	102.23 (14)
C13—C14—H14B	109.5	C2—Co1—C3	108.51 (15)
H14A—C14—H14B	109.5	C1—Co1—P2	152.54 (10)
C13—C14—H14C	109.5	C2—Co1—P2	93.17 (10)
H14A—C14—H14C	109.5	C3—Co1—P2	99.65 (9)
H14B—C14—H14C	109.5	C1—Co1—P1	88.11 (10)
C13—C15—H15A	109.5	C2—Co1—P1	145.53 (10)
C13—C15—H15B	109.5	C3—Co1—P1	104.15 (10)
H15A—C15—H15B	109.5	P2—Co1—P1	70.49 (3)
C13—C15—H15C	109.5	C29—Co2—C28	93.93 (12)
H15A—C15—H15C	109.5	C29—Co2—C30	101.01 (12)
H15B—C15—H15C	109.5	C28—Co2—C30	106.21 (13)
C21—C16—C17	118.6 (2)	C29—Co2—P3	86.94 (8)
C21—C16—P2	120.0 (2)	C28—Co2—P3	145.58 (9)
C17—C16—P2	121.3 (2)	C30—Co2—P3	107.38 (9)
C18—C17—C16	120.1 (3)	C29—Co2—P4	153.27 (9)
C18—C17—H17	120.0	C28—Co2—P4	96.14 (9)
C16—C17—H17	120.0	C30—Co2—P4	99.84 (9)
C19—C18—C17	120.4 (3)	P3—Co2—P4	70.93 (2)
C19—C18—H18	119.8	C58—Co3—C56	105.75 (13)
C17—C18—H18	119.8	C58—Co3—C55	111.89 (14)
C20—C19—C18	119.9 (3)	C56—Co3—C55	108.22 (14)
C20—C19—H19	120.1	C58—Co3—C57	110.19 (14)
C18—C19—H19	120.1	C56—Co3—C57	110.86 (13)
C19—C20—C21	120.3 (3)	C55—Co3—C57	109.85 (14)
C19—C20—H20	119.9	C61—Co4—C60	110.15 (15)
C21—C20—H20	119.9	C61—Co4—C62	111.04 (18)
C20—C21—C16	120.7 (3)	C60—Co4—C62	106.30 (17)
C20—C21—H21	119.7	C61—Co4—C59	109.74 (15)
C16—C21—H21	119.7	C60—Co4—C59	107.83 (17)
C23—C22—C27	119.7 (2)	C62—Co4—C59	111.68 (17)
C23—C22—P2	118.6 (2)	C4—N1—P1	125.26 (19)
C27—C22—P2	120.70 (19)	C4—N1—H1	114 (2)
C24—C23—C22	119.8 (3)	P1—N1—H1	119 (2)
C24—C23—H23	120.1	C13—N2—P2	128.44 (16)
C22—C23—H23	120.1	C13—N2—P1	129.22 (16)
C25—C24—C23	120.2 (3)	P2—N2—P1	96.18 (11)
C25—C24—H24	119.9	C31—N3—P3	126.15 (17)
C23—C24—H24	119.9	C31—N3—H3	113 (2)
C24—C25—C26	119.9 (3)	P3—N3—H3	120 (2)
C24—C25—H25	120.0	C40—N4—P3	130.76 (17)
C26—C25—H25	120.0	C40—N4—P4	126.89 (17)
C27—C26—C25	120.6 (3)	P3—N4—P4	97.02 (10)
C27—C26—H26	119.7	N1—P1—N2	117.14 (12)
C25—C26—H26	119.7	N1—P1—C7	102.57 (12)
C26—C27—C22	119.7 (3)	N2—P1—C7	108.97 (12)
C26—C27—H27	120.1	N1—P1—Co1	116.72 (9)
C22—C27—H27	120.1	N2—P1—Co1	95.18 (8)
O4—C28—Co2	177.9 (3)	C7—P1—Co1	116.75 (9)
O5—C29—Co2	175.8 (2)	N1—P1—P2	123.42 (9)
O6—C30—Co2	177.8 (3)	C7—P1—P2	132.52 (9)
N3—C31—C33	109.4 (2)	Co1—P1—P2	54.48 (2)
N3—C31—C32	111.0 (2)	N2—P2—C22	111.46 (11)
C33—C31—C32	111.8 (2)	N2—P2—C16	109.52 (11)
N3—C31—H31	108.2	C22—P2—C16	104.67 (12)
C33—C31—H31	108.2	N2—P2—Co1	95.80 (8)
C32—C31—H31	108.2	C22—P2—Co1	112.73 (8)
C31—C32—H32A	109.5	C16—P2—Co1	122.45 (9)
C31—C32—H32B	109.5	N2—P2—P1	41.95 (7)
H32A—C32—H32B	109.5	C22—P2—P1	132.29 (9)
C31—C32—H32C	109.5	C16—P2—P1	120.68 (8)
H32A—C32—H32C	109.5	Co1—P2—P1	55.03 (2)
H32B—C32—H32C	109.5	N3—P3—N4	116.68 (11)
C31—C33—H33A	109.5	N3—P3—C34	101.84 (11)
C31—C33—H33B	109.5	N4—P3—C34	108.01 (11)
H33A—C33—H33B	109.5	N3—P3—Co2	116.83 (8)
C31—C33—H33C	109.5	N4—P3—Co2	95.29 (7)
H33A—C33—H33C	109.5	C34—P3—Co2	118.70 (8)
H33B—C33—H33C	109.5	N3—P3—P4	123.80 (8)
C39—C34—C35	119.2 (2)	C34—P3—P4	132.22 (9)
C39—C34—P3	120.7 (2)	Co2—P3—P4	54.69 (2)
C35—C34—P3	120.0 (2)	N4—P4—C43	111.06 (11)
C36—C35—C34	119.9 (3)	N4—P4—C49	106.85 (11)
C36—C35—H35	120.0	C43—P4—C49	104.25 (12)
C34—C35—H35	120.0	N4—P4—Co2	94.80 (7)
C37—C36—C35	120.2 (3)	C43—P4—Co2	115.86 (9)
C37—C36—H36	119.9	C49—P4—Co2	123.18 (9)
C35—C36—H36	119.9	C43—P4—P3	132.93 (9)
C36—C37—C38	120.2 (3)	C49—P4—P3	119.05 (9)
C36—C37—H37	119.9	Co2—P4—P3	54.38 (2)
C38—C37—H37	119.9	C64—C63—C68	120.0
C37—C38—C39	120.5 (3)	C64—C63—C69	119.8 (5)
C37—C38—H38	119.8	C68—C63—C69	120.2 (5)
C39—C38—H38	119.8	C65—C64—C63	120.0
C38—C39—C34	120.0 (3)	C65—C64—H64	120.0
C38—C39—H39	120.0	C63—C64—H64	120.0
C34—C39—H39	120.0	C64—C65—C66	120.0
N4—C40—C41	111.2 (2)	C64—C65—H65	120.0
N4—C40—C42	111.3 (2)	C66—C65—H65	120.0
C41—C40—C42	110.7 (2)	C67—C66—C65	120.0
N4—C40—H40	107.8	C67—C66—H66	120.0
C41—C40—H40	107.8	C65—C66—H66	120.0
C42—C40—H40	107.8	C68—C67—C66	120.0
C40—C41—H41A	109.5	C68—C67—H67	120.0
C40—C41—H41B	109.5	C66—C67—H67	120.0
H41A—C41—H41B	109.5	C67—C68—C63	120.0
C40—C41—H41C	109.5	C67—C68—H68	120.0
H41A—C41—H41C	109.5	C63—C68—H68	120.0
H41B—C41—H41C	109.5	C63—C69—H69A	109.5
C40—C42—H42A	109.5	C63—C69—H69B	109.5
C40—C42—H42B	109.5	H69A—C69—H69B	109.5
H42A—C42—H42B	109.5	C63—C69—H69C	109.5
C40—C42—H42C	109.5	H69A—C69—H69C	109.5
H42A—C42—H42C	109.5	H69B—C69—H69C	109.5
H42B—C42—H42C	109.5		
			
C12—C7—C8—C9	1.2 (4)	C8—C7—P1—Co1	−86.4 (2)
P1—C7—C8—C9	176.7 (2)	C12—C7—P1—P2	154.23 (19)
C7—C8—C9—C10	−0.8 (5)	C8—C7—P1—P2	−21.2 (3)
C8—C9—C10—C11	0.0 (5)	C13—N2—P2—C22	−76.0 (2)
C9—C10—C11—C12	0.3 (5)	P1—N2—P2—C22	130.11 (11)
C8—C7—C12—C11	−0.9 (4)	C13—N2—P2—C16	39.3 (2)
P1—C7—C12—C11	−176.5 (2)	P1—N2—P2—C16	−114.55 (12)
C10—C11—C12—C7	0.1 (5)	C13—N2—P2—Co1	166.8 (2)
C21—C16—C17—C18	−0.8 (4)	P1—N2—P2—Co1	12.91 (9)
P2—C16—C17—C18	−179.6 (2)	C13—N2—P2—P1	153.9 (3)
C16—C17—C18—C19	0.9 (4)	C23—C22—P2—N2	160.8 (2)
C17—C18—C19—C20	−0.2 (4)	C27—C22—P2—N2	−30.9 (2)
C18—C19—C20—C21	−0.7 (4)	C23—C22—P2—C16	42.6 (2)
C19—C20—C21—C16	0.8 (4)	C27—C22—P2—C16	−149.2 (2)
C17—C16—C21—C20	−0.1 (4)	C23—C22—P2—Co1	−92.8 (2)
P2—C16—C21—C20	178.8 (2)	C27—C22—P2—Co1	75.5 (2)
C27—C22—C23—C24	0.5 (4)	C23—C22—P2—P1	−155.44 (17)
P2—C22—C23—C24	168.8 (2)	C27—C22—P2—P1	12.8 (3)
C22—C23—C24—C25	0.0 (5)	C21—C16—P2—N2	−74.9 (2)
C23—C24—C25—C26	0.0 (5)	C17—C16—P2—N2	104.0 (2)
C24—C25—C26—C27	−0.6 (5)	C21—C16—P2—C22	44.7 (2)
C25—C26—C27—C22	1.0 (5)	C17—C16—P2—C22	−136.5 (2)
C23—C22—C27—C26	−1.0 (4)	C21—C16—P2—Co1	174.48 (18)
P2—C22—C27—C26	−169.1 (2)	C17—C16—P2—Co1	−6.7 (3)
C39—C34—C35—C36	−0.8 (4)	C21—C16—P2—P1	−119.9 (2)
P3—C34—C35—C36	−176.6 (2)	C17—C16—P2—P1	59.0 (2)
C34—C35—C36—C37	0.1 (4)	C31—N3—P3—N4	74.6 (2)
C35—C36—C37—C38	0.4 (4)	C31—N3—P3—C34	−168.1 (2)
C36—C37—C38—C39	−0.3 (4)	C31—N3—P3—Co2	−37.1 (2)
C37—C38—C39—C34	−0.3 (4)	C31—N3—P3—P4	26.8 (2)
C35—C34—C39—C38	0.9 (4)	C40—N4—P3—N3	42.9 (3)
P3—C34—C39—C38	176.7 (2)	P4—N4—P3—N3	−111.83 (11)
C48—C43—C44—C45	−1.0 (4)	C40—N4—P3—C34	−71.0 (2)
P4—C43—C44—C45	−175.6 (2)	P4—N4—P3—C34	134.29 (11)
C43—C44—C45—C46	−0.2 (5)	C40—N4—P3—Co2	166.5 (2)
C44—C45—C46—C47	0.7 (5)	P4—N4—P3—Co2	11.80 (9)
C45—C46—C47—C48	0.0 (5)	C40—N4—P3—P4	154.8 (3)
C46—C47—C48—C43	−1.3 (4)	C39—C34—P3—N3	34.8 (2)
C44—C43—C48—C47	1.8 (4)	C35—C34—P3—N3	−149.4 (2)
P4—C43—C48—C47	176.3 (2)	C39—C34—P3—N4	158.2 (2)
C54—C49—C50—C51	−0.9 (4)	C35—C34—P3—N4	−26.0 (2)
P4—C49—C50—C51	173.6 (2)	C39—C34—P3—Co2	−95.0 (2)
C49—C50—C51—C52	0.2 (4)	C35—C34—P3—Co2	80.8 (2)
C50—C51—C52—C53	0.2 (4)	C39—C34—P3—P4	−161.90 (16)
C51—C52—C53—C54	0.3 (4)	C35—C34—P3—P4	13.9 (3)
C52—C53—C54—C49	−1.0 (4)	C40—N4—P4—C43	72.0 (2)
C50—C49—C54—C53	1.4 (4)	P3—N4—P4—C43	−131.83 (11)
P4—C49—C54—C53	−173.4 (2)	C40—N4—P4—C49	−41.1 (2)
C6—C4—N1—P1	94.1 (3)	P3—N4—P4—C49	115.07 (12)
C5—C4—N1—P1	−141.2 (3)	C40—N4—P4—Co2	−167.91 (19)
C14—C13—N2—P2	−168.3 (2)	P3—N4—P4—Co2	−11.74 (9)
C15—C13—N2—P2	70.0 (3)	C40—N4—P4—P3	−156.2 (3)
C14—C13—N2—P1	−22.7 (3)	C44—C43—P4—N4	−152.1 (2)
C15—C13—N2—P1	−144.4 (2)	C48—C43—P4—N4	33.4 (2)
C33—C31—N3—P3	135.5 (2)	C44—C43—P4—C49	−37.4 (2)
C32—C31—N3—P3	−100.6 (3)	C48—C43—P4—C49	148.1 (2)
C41—C40—N4—P3	137.6 (2)	C44—C43—P4—Co2	101.3 (2)
C42—C40—N4—P3	13.7 (3)	C48—C43—P4—Co2	−73.3 (2)
C41—C40—N4—P4	−74.4 (3)	C44—C43—P4—P3	165.62 (17)
C42—C40—N4—P4	161.70 (19)	C48—C43—P4—P3	−8.9 (3)
C4—N1—P1—N2	−71.6 (3)	C50—C49—P4—N4	−101.6 (2)
C4—N1—P1—C7	169.2 (2)	C54—C49—P4—N4	73.0 (2)
C4—N1—P1—Co1	40.2 (3)	C50—C49—P4—C43	140.7 (2)
C4—N1—P1—P2	−23.2 (3)	C54—C49—P4—C43	−44.6 (2)
C13—N2—P1—N1	−42.8 (3)	C50—C49—P4—Co2	6.0 (3)
P2—N2—P1—N1	110.82 (13)	C54—C49—P4—Co2	−179.38 (17)
C13—N2—P1—C7	73.0 (2)	C50—C49—P4—P3	−58.3 (2)
P2—N2—P1—C7	−133.40 (12)	C54—C49—P4—P3	116.3 (2)
C13—N2—P1—Co1	−166.4 (2)	C68—C63—C64—C65	0.0
P2—N2—P1—Co1	−12.81 (9)	C69—C63—C64—C65	177.7 (6)
C13—N2—P1—P2	−153.6 (3)	C63—C64—C65—C66	0.0
C12—C7—P1—N1	−39.8 (3)	C64—C65—C66—C67	0.0
C8—C7—P1—N1	144.7 (2)	C65—C66—C67—C68	0.0
C12—C7—P1—N2	−164.6 (2)	C66—C67—C68—C63	0.0
C8—C7—P1—N2	19.9 (3)	C64—C63—C68—C67	0.0
C12—C7—P1—Co1	89.1 (2)	C69—C63—C68—C67	−177.7 (6)

### Hydrogen-bond geometry (Å, º)

**Table:**

*D*—H···*A*	*D*—H	H···*A*	*D*···*A*	*D*—H···*A*
N1—H1···O10^i^	0.87 (1)	2.22 (2)	3.041 (3)	159 (3)
N3—H3···O13^ii^	0.86 (1)	2.27 (1)	3.101 (3)	163 (3)

Symmetry codes: (i) −*x*+1/2, *y*+1/2, −*z*+1/2; (ii) −*x*+1, −*y*+1, −*z*+1.
